# The effect of supplementary ultraviolet wavelengths on broiler chicken welfare indicators

**DOI:** 10.1016/j.applanim.2018.10.002

**Published:** 2018-12

**Authors:** Charlotte James, Lucy Asher, Katherine Herborn, Julian Wiseman

**Affiliations:** a Department of Animal Sciences, University of Nottingham, Sutton Bonington Campus, Leicestershire, UK; b Centre for Behaviour and Evolution IoN, Newcastle University, Henry Wellcome Building, Newcastle, UK

**Keywords:** Broiler chicken, Poultry welfare, Husbandry, Lighting, Ultraviolet light

## Abstract

Qualities of the light environment are important for good welfare in a number of species. In chickens, UVA light is visible and may facilitate flock interactions. UVB wavelengths promote endogenous vitamin D synthesis, which could support the rapid skeletal development of broiler chickens. The aim of the study was to investigate the impacts of Ultraviolet wavelengths (UV) on welfare indicators in broiler chickens.

Day-old Ross 308 birds reared under commercially representative conditions were randomly assigned to one of three lighting treatments: A) White Light Emitting Diode (LED) and supplementary UVA LED lighting (18-hour photoperiod); B) White LED with supplementary UVA and UVB fluorescent lighting providing 30 micro watts/cm^2^ UVB at bird level (on for 8 h of the total photoperiod to avoid over-exposure of UVB); C) White LED control group, representative of farm conditions (18-hour photoperiod).

Welfare indicators measured were; feather condition (day 24, n = 546), tonic immobility duration (day 29, n = 302), and gait quality, using the Bristol Gait Score (day 31, n = 293). Feather condition was improved in male broilers in the UVA treatment (A), compared to the control treatment (C). Birds in the UVA treatment had shorter tonic immobility durations compared to the control treatment (C), suggesting lower fearfulness. Broilers reared in UVA (A) and UVA + UVB (B) had better Bristol Gait Scores compared to the control (C).

Together these results suggest UV may be beneficial for broiler chicken welfare. While treatment A and B both provided UVA, the improvements in welfare indicators were not consistent, which may be due to exposure time-dependent beneficial effects of UVA.

The modification of commercial lighting regimes to incorporate UVA wavelengths for indoor-reared broiler chickens would be an achievable change with significant positive impacts on bird welfare.

## Highlights

• Supplementary UVA wavelengths improved feather condition in male broiler chickens.
• UV wavelengths reduced fear responses (tonic immobility duration) in broilers.
• UV wavelengths improved walking ability (Bristol gait Score) in broiler chickens.
• UV wavelengths have potential to improve broiler chicken welfare.

## 1 Introduction

Qualities of lighting are known to be important for welfare in a range of species (McLennan and Taylor-Jeffs, 2004; Migaud et al., 2007; Oliveira and Lara, 2016; Taylor et al., 2006). Aspects of the lighting environment found to be important for welfare include the length of the photoperiod and scotoperiod for the maintenance of circadian rhythms, the light intensity and the wavelength composition of the light source (Campo and Davila, 2002; Deep et al., 2013; Fuller et al., 2016). Birds have different visual capacities and spectral sensitivities to humans and are able to perceive UVA wavelengths invisible to the human eye (Goldsmith, 2006; Waldvogel, 1990). Many birds, including domestic poultry, also possess feathers that reflect UVA wavelengths (Bartels et al., 2017; Mullen and Pohland, 2008; Prescott and Wathes, 1999b). The presence or absence of UV wavelengths and the UV reflective properties of bird’s feathers influence foraging behaviour (Passerines: Church et al., 1998; Honkavaara et al., 2004; Galliformes: Siitari et al., 2002), mate selection (Psittacines: Griggio et al., 2010; Passerines: Maddocks et al., 2002), nestling resource allocation (Apodiformes: Bize et al., 2006; Passerines: Jourdie et al., 2004; Tanner and Richner, 2008), and the recognition of brood-parasite eggs (*Acrocephalus scirpaceus*: Šulc et al., 2016). The domestic fowl is sensitive to UVA wavelengths as low as 360 nm (Prescott and Wathes, 1999a; Osorio et al., 1999). Despite this, industry standard housing for broiler chickens (bred commercially for meat production) in the UK is indoor housing with no exposure to UV or natural light throughout the whole rearing period.

While windows may be incorporated into poultry houses, glass does not typically transmit any UVB wavelengths of light and limits the transmission of UVA wavelengths depending on the type of glass used (Duarte et al., 2009). Consequently, light from windows may not be representative of sunlight or appear “natural” to a chicken. In this paper the potential of artificial UVA and UVB wavelengths for improving broiler chicken welfare is explored.

Exposure to UV in sunlight stimulates beta-endorphin production in humans creating a sense of well-being and relaxation, relieving pain and promoting wound healing (Slominski et al., 2012; Sprouse-Blum et al., 2010). While the subjective emotional responses of animals in response to UV is hard to determine, their preferences when offered choices between different lighting environments, or changes in behavioural expression in different light environments, can be measured and used to make judgments about how their welfare is affected. Widowski et al. (1992) found laying hens had a preference for fluorescent light over incandescent light; Prayitno et al. (1997a) found broilers reared for 28 days under a single light colour showed a preference for blue wavelengths when subsequently offered a choice between blue, green, red or white light; with the exception of those raised in blue light, which preferred the novel green light environment. Another study by Prayitno et al. (1997b) found broiler chickens were more active and aggressive in red light and were calmer in blue light.

Given the importance of UVA as a component of avian visual feedback, the provision of these wavelengths could be considered a valuable form of environmental enrichment (EE), using the definition proposed by Shepherdson (1998) of: “An animal husbandry principle that seeks to enhance the quality of captive animal care by providing the environmental stimuli necessary for optimal psychological and physiological well-being’’.

Few studies have assessed the impacts of artificial lighting regimes including UV wavelengths on the welfare and behaviour of chickens.

Ruis et al. (2010) conducted a series of experiments in laying hens reared under different optimised lighting conditions and found several positive outcomes with UVA: increased preening and ground pecking, reduced fearful behaviour and reduced gentle feather pecking. Similarly, Kristensen et al. (2007) showed that six-week old broiler chickens performed more preening, object manipulation, foraging, and walking when reared in lighting conditions that included UVA. Maddocks et al. (2001) found significantly lower baseline levels of corticosterone in chicks along with a non-significant trend for increased exploratory behaviours when provided with UVA.

However, not all outcomes were positive: when laying hens were reared to 50 weeks, Ruis et al. (2010) found that UVA increased incidence of severe feather pecking at certain ages. This was reduced in all lighting treatments after the introduction of substrate (Ruis et al., 2010). Therefore, Ruis et al. (2010) proposed that UVA may have made the feathers of conspecifics look more appealing than in standard lighting, attracting more severe pecking in an environment lacking other stimuli. This idea is also supported by results of Sherwin et al. (1999) who observed reductions in injurious pecking in turkey poults reared with supplementary UVA in conjunction with other EE. Interestingly, Jones et al. (2001) found broiler breeder hens spent longer observing cockerels illuminated with UVA and mated more frequently compared to broiler breeders in standard lighting conditions, supporting this idea of enhanced interest in feathering, and again emphasising the importance of considering the impact of age or maturity. Jones et al. (2001) found UVA illumination was also associated with increased locomotion which may have positive impacts on walking ability (Foutz et al., 2007b).

Together, the literature reviewed suggest that whilst UVA provision alone may not be a “quick fix” for welfare problems such as feather pecking, UVA may improve the quality and potentially the reliability of visual feedback as perceived by poultry, enhancing the appearance of both conspecifics and their environment. The studies discussed above suggest that unless animals are housed in otherwise impoverished environments, UVA wavelengths could potentially facilitate more harmonious flock interactions and promote the expression of natural behaviours. Floor-housed broiler chickens are therefore a good candidate for investigating the impacts of UV wavelengths on welfare, as they are typically provided with substrate and have a short rearing period before the onset of maturity. To date, no studies have assessed the value of UVA and UVB wavelengths in conjunction with LED lighting systems.

UVB wavelengths (290–320 nm), while not visible to chickens, may offer health and welfare benefits through supporting the endogenous synthesis of vitamin D, which plays an important and well-established role in calcium metabolism (Stanford, 2006; Matos, 2008). Non-infectious skeletal deformities represent a significant welfare problem in commercially reared broiler chickens (Knowles et al., 2008). Lameness is predominantly associated with selection for rapid growth rates (Julian, 1998), though may be influenced by both genetic and environmental parameters including disease status, flock management and nutritional deficiencies, Including Vitamin D deficiencies (Kapell et al., 2012; Waldenstedt, 2006). Both dietary vitamin D supplementation (Ledwaba and Roberson, 2003; Whitehead et al., 2004; Gómez-Verduzco et al., 2013), and UVB wavelength provision have been found to support the skeletal development and bone mineralisation of chicks (Edwards, 2003; Fleming, 2008; Tian et al., 1994). As such, UVB provision may lead to improvements in walking ability and subsequently allow birds to feed more and engage in natural behaviours that would otherwise be prohibitively energetically expensive (Weeks et al., 2000).

In the current study three welfare indicators were measured to investigate the effects of UVA and UVB wavelengths on ROSS 308 broiler chickens: feather condition, tonic immobility and gait score. Feather condition was assessed as the growth of feathers is important in commercial settings to provide birds with protection from injury and for thermoregulation (Leeson and Walsh, 2004a; Leeson and Walsh, 2004b). Feather growth and feather quality are impaired by both exogenous administration and environmental stress-induced endogenous production of corticosterone (*Sturnus vulgaris* DesRochers et al., 2009; Lattin et al., 2011). Plumage condition has also been associated with indicators of stress and fearfulness such as tonic immobility duration and blood leukocyte ratios (Campo et al., 2001, 2007; Campo and Prieto, 2009). This makes feather condition an interesting parameter to investigate in conjunction with other welfare measures.

Tonic Immobility (T.I) duration has been proposed as a useful measure of fearfulness, an adaptive anti-predator response which is increased in more fearful birds (Gallup, 1979; Jones and Faure, 1981). Broiler chickens exhibiting shorter tonic immobility duration have been found to have improved growth performance and higher adaptability to stress (Wang et al., 2013). T.I duration is responsive to circulating stress hormones and increases following corticosterone administration (Jones et al., 1988) or in response to stressors such as continuous lighting (Campo et al., 2007) or noise (Campo et al., 2005). T.I duration is also shorter in birds provided with environmental enrichment (Jones and Waddington, 1992), and thus it would be predicted that UVA wavelengths may reduce tonic immobility duration.

Lastly the Bristol Gait Score developed by Kestin et al. (1992) is a validated scoring system used to evaluate the walking ability of broiler chickens. Higher scores where mobility is compromised are indicative of poor welfare. The provision of UVB wavelengths may support skeletal development and bone mineralisation, (Edwards, 2003; Fleming, 2008; Tian et al., 1994) potentially leading to improvements in walking ability. Similarly, as UVA has been shown to encourage activity in broilers, the increased mechanical loading of the skeleton associated with higher activity levels may contribute to improvements in walking ability (Foutz et al., 2007b).

The aim of the study was to investigate the impact of UVA and UVB wavelengths on the feather condition, fearfulness and walking ability of floor-housed broiler chickens. It was hypothesised that UVA provision would reduce fearfulness and that both UVA and UVB provision could potentially improve walking ability.

## 2 Methods

### 2.1 Animals and husbandry

The current study used 638 Ross 308 broiler chickens obtained from P D Hook Hatcheries Limited, UK on hatch day. Chicks were from a 35-45-week-old parent flock and received vaccinations for Infectious Bronchitis at the hatchery. On arrival chicks were weighed and randomly assigned to one of six temperature-controlled rooms, each containing a single pen measuring 3.4 m x 2.5 m (n = 106–107 chicks per pen / n = 212–213 chicks per lighting treatment). The arrival of the birds was staggered with pen one, two and three (Flock one) arriving a week prior to pen four, five and six (Flock two).

Birds were fed *ad. libitum* on a commercial wheat-based diet provided by ABN, AB Agri, UK, and reared on a bedding of wood shavings. Fresh bedding was added if litter appeared wet. Each pen had a small bale of straw for enrichment purposes. The final stocking density reached by the end of the trial was a commercially representative 33 kg/m^2^ based on a total useable floor area per pen of 7 m^2^ after subtracting space for feeders, drinkers and enrichment bales. All broilers were individually identified with wing tags at 7 days old.

All birds were individually weighed in a large bucket using electronic scales at 27 days old.

At 9, 21 and 30 days old six birds per pen were culled to assess their development. Final depletion took place over 5 days when birds were 35 (Flock one only) 41 (Flock 2 only) 42, 43, 44 and 45 days old. All birds were euthanised using an overdose of Pentobarbital Sodium via the intraperitoneal route for 9 day-old chicks or by intravenous wing vein injection for all other ages. All birds were sexed post-mortem by the identification of testes or ovaries, (Females n = 293, Males n = 287, unsexed = 8). There were 50 mortalities during the trial which were also unsexed: Treatment A, n = 7, Treatment B, n = 17, Control, n = 26, (These figures include birds culled for health reasons).

Standard biosecurity measures were in place governing entry of personnel and the experiment was reviewed and authorised by the Animal Welfare and Ethics Reviewing Body at the University of Nottingham, UK.

### 2.2 Lighting treatments

**Fig. 1:**
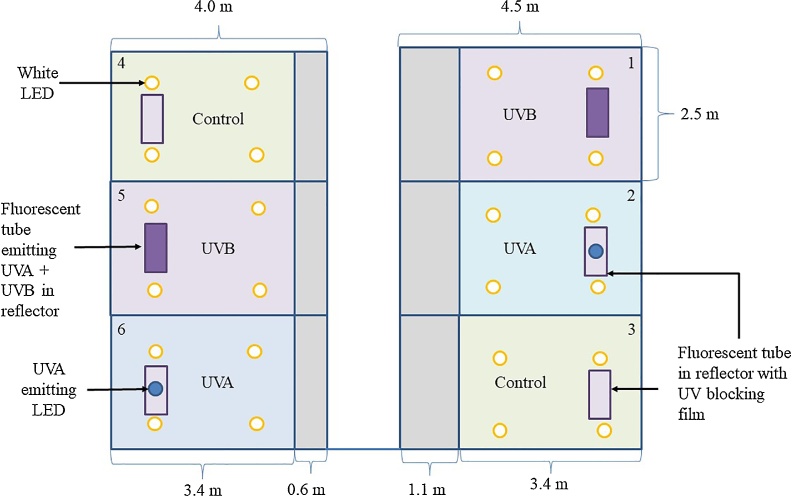
Experimental set up. There were six pens of equal dimensions, each in a separate temperature-controlled room. Lighting treatments were replicated across two pens. The main light source was white Light Emitting Diodes (LEDs), with supplementary UVA wavelengths (Provided by an additional UVA LED for 18 h) or supplementary UVA + UVB (Provided by an additional fluorescent light for 8 h). Fluorescent lights were fitted with UV blocking film in the UVA only treatment and the control treatment.

There were three treatments in the current experiment; (A) UVA wavelengths but no UVB, (B) including both UVA and UVB wavelengths and the control (C) with no UV wavelengths, representative of commercial practice. Each treatment was replicated across two pens. The main light source used in all pens for this experiment was the Agricultural Lighting Induction System (ALIS) which consisted of 4 × 8 W clip-on LEDs provided by Greengage Lighting Ltd (Edinburgh,UK), installed 170 cm from the ground. All pens were fitted with a single 18 W 12% UVB D3 + T8 florescent light (Arcadia Products plc, Surrey, UK) in a reflector, powered by a high frequency 18 W electronic ballast (Komodo, Leicestershire, UK) (Fig. 1).

The fluorescent light provided the UVA and UVB wavelengths for treatment B, and was suspended from a length of steel cable, secured using cable-ties, at a height of 50 cm from the ground to provide 30 μW/cm² of UVB at chick head height when measured with a UVB meter (Solarmeter® Model 6.2, Pennsylvania, USA). The height of the fluorescent lamp was altered by attaching further cable ties to shorten the length of wires suspending the lamp as the chickens grew, and the corresponding lamp height was replicated across the other treatments.

**Fig. 2:**
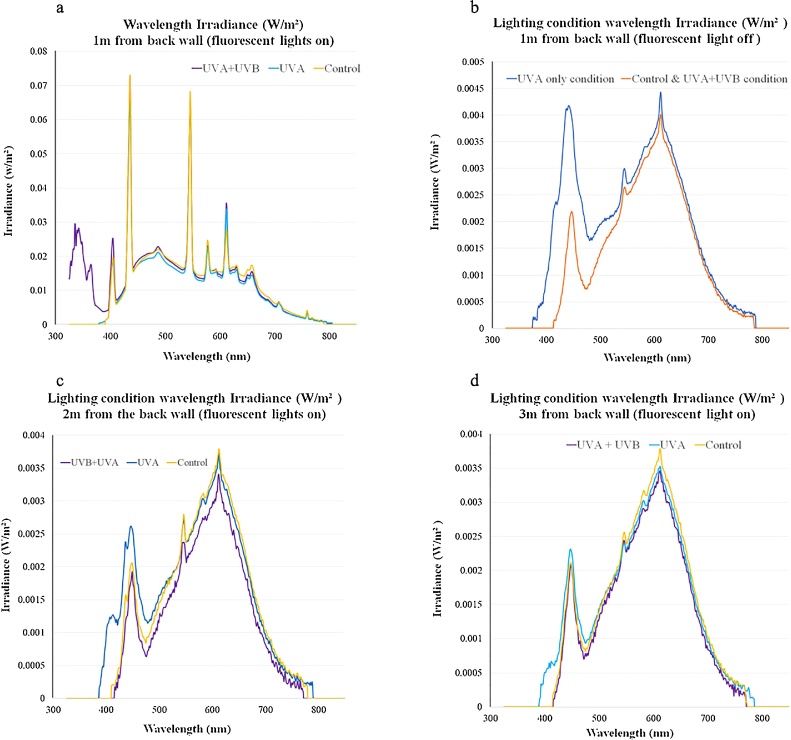
Spectral composition of the lighting treatments. Fig. 2.a and b show the mean spectroradiometry measurements 25 cm from the ground 1 m from the back wall (directly under the fluorescent light). Fig. 2.a shows data when the fluorescent lights are switched on (for 8 h of the total 18-hour photoperiod) or switched off (Fig. 2.b). These lamps provide UVA and UVB wavelengths in treatment B but are blocked by a clear filter in treatment A (UVA only) and treatment C (control). The UVA wavelengths in treatment A are provided by a UVA LED, which also increases the amount of violet and blue visible light (Fig. 2.b). Fig. 2.c and. d show the mean spectroradiometry measurements for the lighting conditions taken two (Fig. 1.c) or three (Fig. 1.d) meters from the back wall of each pen 25 cm from the ground when the fluorescent lights are on. The UVA provided in treatment A by the UVA LED extended across the whole pen, whereas the UVA and UVB provided in treatment AB by the fluorescent light is much more localised and undetectable a meter away from the fluorescent lamp. Thus, broiler chickens could self-select their exposure to UV in treatment B.

It was necessary to fit these fluorescent lights in all pens as they create a localised patch of higher light intensity with a spectral output distinct from the ALIS LEDs (as seen in Fig. 2.a–b). However, in treatments C and A the fluorescent lights were fitted with clear CON-TROL-CURE® UV Blocking films (Epak Electronics, Somerset UK). No UVB was measured in treatment A or C using the Solarmeter. A single clip on UVA LED (Greengage Lighting Ltd, Edinburgh,UK) was added to the ALIS in treatment A to provide UVA wavelengths.

Prior to the introduction of the birds to the pens, the light conditions of all pens were measured with a spectroradiometer (Model,FieldSpec® HandHeld 2 with a wavelength range of 325–1075 nm and an accuracy ± 1 nm, ASD inc. Colorado, USA). Spectrometry readings were taken along the midline of each pen at 1, 2 and 3 m from the back wall at a height of 25 cm from the ground. Raw data were extracted using ViewSpec™ Pro software (ASD inc. Colorado, USA) and light intensity was calculated in “clux” as described by Nuboer et al (1992) and Prescott and Wathes (1999a) to ensure light intensity was approximately the same (when adjusted to the spectral sensitivity of the chicken) across all conditions.

The mean clux measurements obtained 1, 2 and 3 m from the back wall when the fluorescent lights were switched on were: 178.4 SEM 10.7, 19.0 SEM 0.8 and 19.0 SEM 0.5. The Irradiance (W/m^2^) of wavelengths (nm) in each treatment are shown in Figs. 2.a–d.

The same photoperiod was maintained across all lighting treatments. The ALIS system was controlled by an automated DTD (Dusk till Dawn) Lighting Processor Control, (Greengage Lighting Ltd), which incorporates 30 min of “dawn” and “dusk” dimming at either end of the programmed photoperiod. The scotoperiod was programmed to start at 11 pm as single hour of darkness on the day the chicks arrived, increasing by an hour each night, until 6 h of consecutive darkness was achieved (11 pm-5am). The fluorescent lights were controlled by mechanical timers, (Maplin, Rotherham, UK) programmed to switch on from 5:30-9:30am and 4:30-8:30pm for a total of 8 h of the 18 h photoperiod. The fluorescent lights were not left on for the whole photoperiod to reduce the risk of overexposure of UVB (Moan et al., 2013; Yam and Kwok, 2014). However, this meant UVA was provided for the whole photoperiod of 18 h in treatment A (via the UVA LED), but only for 8 h in treatment B (via the fluorescent light with no filter).

### 2.3 Feather score

The feather condition of all the birds (n = 546) was assessed when they were 24 days old using the RSPCA feather score index. This is a four-point scale of 0, 0.5, 1, 1.5 and 2 assigning birds a score of feather coverage from a score of “full and even over body and wings” (score of 0) to “bare on the body and patchy on the wings” (score of 2;) (RSPCA, 2013).

### 2.4 Tonic immobility duration

T.I duration was measured from 50 to 53 birds per pen (n = 302; treatment A: n = 101, treatment B: n = 101, treatment C: n = 100) at 29 days of age. An area of the pen was sectioned off with opaque boards to allow birds to be individually assessed out of sight of their flock mates. Each bird was gently restrained on their right side on a changing mat, which could be wiped down between birds if needed. The bird was gently restrained with the left wing held closed against the body for 30 s to induce T.I. This was attempted a maximum of three times, after which a score of zero was awarded. The duration of T.I was timed using a digital stopwatch for a maximum duration of 180 s, after which any birds remaining in T.I were gently righted and returned to the main flock.

### 2.5 Walking ability

Walking ability was assessed for n = 293 birds when they were 31 days old. All birds were observed by the same two handlers, who agreed upon a score based on the Bristol Gait Score criteria established by Kestin et al. (1992). The Bristol Gait Score is based on a six - point scale from a score of zero (describing smooth fluid locomotion) to a score of five (where the bird is unable to move). A score of three or higher is considered indicative of compromised welfare and commercially birds obtaining these scores are culled (also in this study). No birds had been culled due to compromised walking ability before gait scoring was carried out, though a total of 4 birds obtained scores of 3 or higher on the day of gait assessment and were culled. The front of each pen was sectioned off with opaque boards to create a runway of 2.5 m. Each individual bird was placed at the end of the runway and encouraged to walk away from the handler to the other side of the pen where a gap was left to allow the bird to re-join the flock.

### 2.6 Statistical analysis

Due to lack of variation in the results of female broilers (n = 9 scores of ≥1) only males’ feather scores were analysed (n = 245 treatment A: n = 21, treatment B: n = 112 treatment C: n = 112,). Only 19 males obtained feather scores of 1.5, so these scores were combined with scores of 1 in to a single category, giving a binary outcome of birds scoring 0.5 (better feathered) or ≥1 (worse feathered).

As T.I duration data were not normally distributed, each bird was assigned a category from 1 to 4 where T.I was: 1) not induced; 2) induced with a time of 1–89 secs; 3) induced with a time of 90–179 secs or; 4) was induced with the maximum time of 180 ss.

A single outlier was removed from the walking ability data set (one bird in the control treatment which received a gait score of 4 on the day of gait assessment).

Generalised linear models (glm) or Ordinal logistic regressions (polr) analysis (polr) was performed in R statistical software. Models were fitted to investigate the impacts of multiple independent variables (sex, weight, lighting treatment, flock, time of T.I test, and handler inducing T.I,) on the following dependent variables: Feather score (glm), gait score (polr) T.I category (polr), likelihood of obtaining the maximum T.I category (glm; binary outcome), and T.I Induction attempts (polr; recorded for n = 272 tests).

“Flock” was not included in the feather score model due to the small sample size of males in Flock 2, Treatment A (n = 2), but was retained in all other models to control for data being collected at different time points as Flock 1 and Flock 2 were a week apart in age.

Backwards elimination was used to exclude variables, based on whether a significant change in model fit (chi-squared test). The final feather score model included only “Lighting treatment” and “Weight at 27 days old”. Final models for T.I duration, likelihood of obtaining the max T.I duration and T.I induction attempts, included only “Treatment” and “Flock”. The final gait score model included “Treatment”, “Flock”, “Weight at 27 days old” and an interaction effect between “Weight” and “Treatment”.

## 3 Results

### 3.1 Feather score

**Fig. 3:**
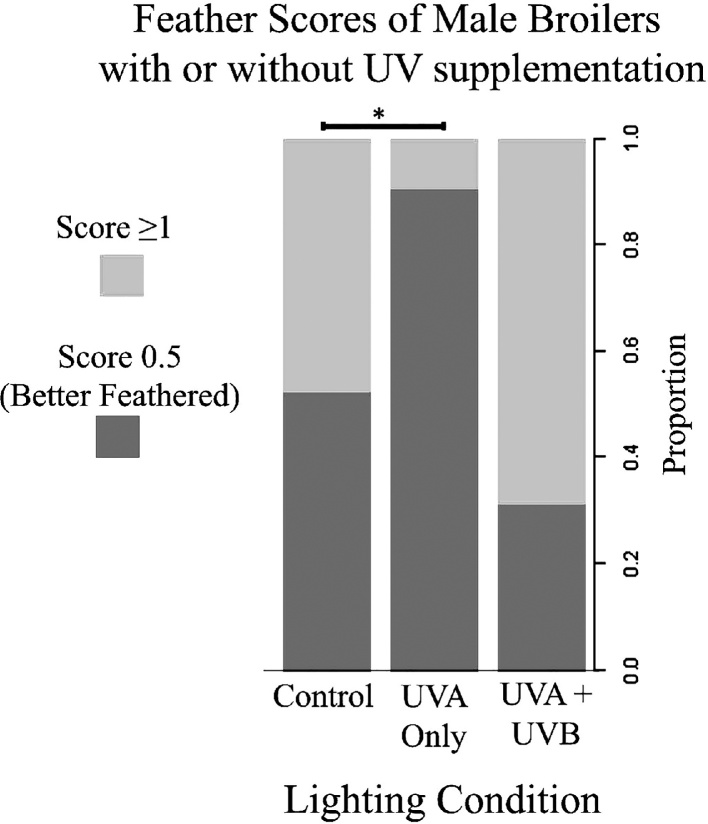
Feather scores of male broiler chickens with or without UV wavelength supplementation. Proportion of male broiler chickens in each lighting treatment obtaining scores of 0.5 (better feathering) or ≥1 (worse feathering). Significant (p < 0.05) differences between treatments are indicated (*).

Twenty four day-old male broiler chickens had significantly better feathering in the UVA only treatment (A, glm: n = 245, z = -2.16, p = 0.031) compared to the control treatment (C, Fig. 3). There was a trend for males to have worse feather scores in the UVA + UVB treatment (B, glm: n = 245, z = 1.85, p = 0.065). Weight also had a significant impact on feather score, with heavier males having poorer feathering than lighter males (glm: n = 245, z = 4.05, p < 0.001). Table 1 shows odds ratios and confidence intervals for feather scores.

**Table 1:** The effect of supplementary ultravioloet wavelengths on Broiler chicken welfare indicators. Odds ratios and 95% confidence intervals are presented for all modelled welfare indicators included in ordered logistic regression ^1^ or generalised linear models^2^.

		Odds ratio	95% confidence intervals		p
Male Feather Scores²			–	+	
Control vs UVA		0.180	0.038	0.851	0.031
Control vs UVA + UVB		1.731	0.967	3.101	0.065
Weight (g)		1.004	1.002	1.006	< 0.001
Tonic Immobility Duration					
Odds of being in a higher T.I category (1-4)¹					
Control vs UVA		0.487	0.293	0.810	0.006
Control vs UVA + UVB		0.647	0.388	1.079	0.095
Flock 1 vs Flock 2		1.078	0.714	1.628	0.721
Odds of obtaining max time (180 sec)²					
Control vs UVA		0.324	0.160	0.654	0.002
Control vs UVA + UVB		0.561	0.298	1.056	0.073
Flock 1 vs Flock 2		1.364	0.786	2.367	0.269
Odds of requiring multiple T.I inductions¹					
Control vs UVA		1.983	1.109	3.545	0.021
Control vs UVA + UVB		1.457	0.854	2.486	0.168
Flock 1 vs Flock 2		0.944	0.594	1.502	0.809
Gait Score¹					
Control vs UVA		0.001	0.001	0.001	< 0.001
Control vs UVA + UVB		0.053	0.051	0.054	< 0.001
Weight (g)		1.005	1.004	1.005	< 0.001
Flock 1 vs Flock 2		2.083	1.293	3.355	0.002
Weight effect (UVA only)		1.004	1.004	1.005	< 0.001
Weight effect (UVA + UVB)		1.001	1.001	1.002	< 0.001

### 3.2 Tonic immobility duration

**Fig. 4:**
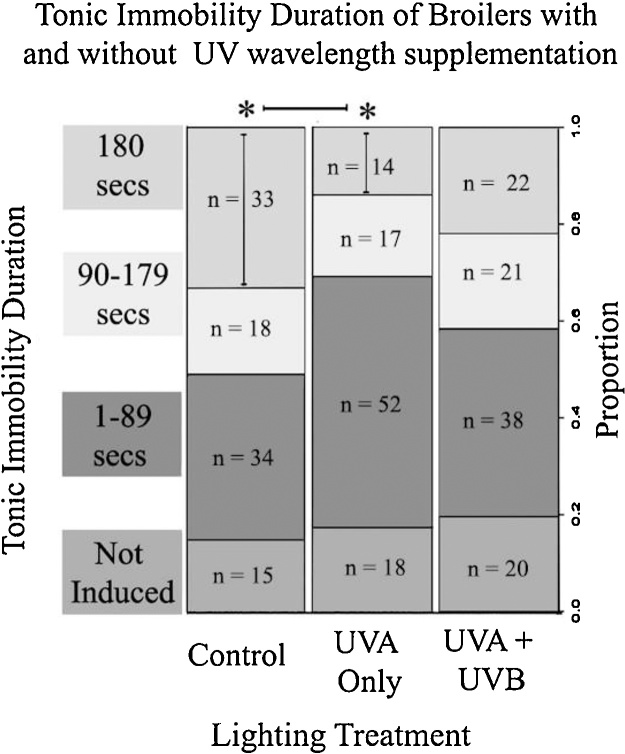
Tonic immobility duration of broiler chickens with or without UV wavelength supplementation. Proportion of broiler chickens in different Tonic Immobility (T.I) categories for each lighting treatment. Significant (p < 0.05) differences between treatments are indicated (*) for both overall T.I duration and the likelihood of obtaining the maximum duration of 180 s.

Broiler chickens in the UVA only treatment had shorter T.I duration than the control group (polr: n = 302, z = -2.77 p = 0.006; Fig. 4). There was also a trend for birds in the UVA + UVB treated group to have shorter T.I duration than the control treatment (polr: n = 302, z= -1.68, p = 0.095). Fewer birds in the UVA only condition obtained the maximum time of 180 ss (glm: n = 302, z= -3.14, p = 0.002) and there was a similar trend in the UVA + UVB treated group (glm: n = 302, z=-1.79, p = 0.073). Birds in the UVA treated group were also more likely to require multiple T.I induction attempts than the control group (polr: n = 272, z = 2.19, p = 0.021). There was no significant effect of different handlers, the time of day the test was performed, sex, weight or flock on T.I induction or duration. Table 1 shows odds ratios and confidence intervals for tonic immobility.

### 3.3 Walking ability

**Fig. 5:**
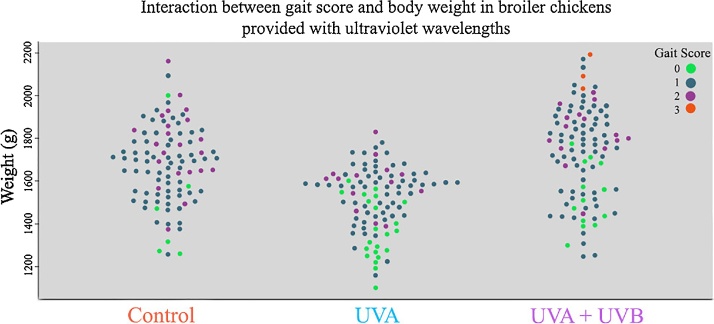
Live weight and walking ability of broiler chickens with or without UV wavelength supplementation. Distribution of broiler chicken weights at 27 days old (g) and corresponding Bristol Gait Score at 31 days old ranging from 0 (fluid locomotion) to 5 (unable to walk) for each treatment.

Broiler chickens with UV wavelength supplementation had improved walking ability compared to birds in the control treatment (Fig. 5). Gait Scores were significantly lower (better) in the UVA + UVB treatment (B, polr: n = 293, z = -229.32, p < 0.001) and the UVA only treatment (A, polr: n = 293, z = -1158.18, p < 0.001). Heavier birds had higher (worse) gait scores (polr: n = 293, z = 24.21, p= <0.001), and there was a significant interaction between weight and treatment. Heavier birds in the UVA only (A vs. C: polr: n = 293, z = 20.86, p= <0.001) and the UVA + UVB treatment (B vs. C polr: n = 293, z = 7.13, p= <0.001) had lower gait scores than control birds of similar weights. Table 1 shows odds ratios and confidence intervals for gait score.

## 4 Discussion

Findings presented here suggest UVA and UVB may offer potential welfare benefits to indoor reared broilers. UVA led to reduced fearfulness, improved walking ability and improved feather condition in male broilers. In the UVA + UVB treatment walking ability was also improved and there was a trend for reduced fearfulness.

### 4.1 Feather cover

Feathering analyses were limited to males, due to lack of variation in female feather scores. The feather development of female broiler chickens is faster than males, which may explain the sex differences observed in the current study (Deschutter and Leeson, 1986; Hancock et al., 1995; Moran, 1981; Siegel, 1963; Wecke et al., 2017). Additionally, the RSPCA feather score, as a general assessment of whole body feather cover, may not be sensitive enough to detect the smaller variations in feather cover that may have been present in females.

Male broilers provided with UVA for the full 18-hour photoperiod had improved feather condition compared to control broilers at 24 days of age. However, there was a non-significant trend for feather condition to be worse in birds provided with only 8 h of UVA + UVB. This could be due to the differences in the length of UVA exposure time, or the more limited distribution of UV wavelengths across the UVA + UVB treatment pen, where UV exposure was localised to an area under the fluorescent lamp, with no UV measured 1 m away from the lamp as shown in Section 2.2, Fig. 2. The possibility that UVB had an inhibitory effect on feather development cannot be ruled out, though no studies currently support or refute this possibility.

There are three reasons why our feathering results are of particular interest. First, feather growth is energetically expensive. The rate feather growth is highest during the first 6 weeks of age (Moran, 1981; Stilborn et al., 1994), with feathers maturing earlier than other body components (Bonato et al., 2016; Gous et al., 1999). Feather growth has been shown to be maintained in preference to, or even at the expense of, muscle development in turkeys when feed availability was restricted (Wylie et al., 2001). As such, under natural conditions feather growth and the maintenance of feather quality is energetically costly and thought to be an indicator of an individual’s condition (Falconiformes: Bortolotti et al., 2002; Passerines: Bulluck et al., 2017; Hill and Montgomerie, 1994; Review: Jovani and Rohwer, 2017), and the quality of their environment (Charadriiformes: Patterson et al., 2015; Will et al., 2014; Passerines: DesRochers et al., 2009; Lattin et al., 2011; Swaddle and Witter, 1994). Aspects of plumage condition are thought to act as honest signals in a variety of social contexts including mate selection (Passerines: Hill, 1990, 1991; Siefferman et al., 2005) signals of social status (*Passer domesticus:* Nakagawa et al., 2007) and parent-offspring communications (Passerines: Tanner and Richner, 2008; Psittaciformes: Griggo et al., 2009). Therefore, our results suggest not only that UVA exposure better enabled individuals to meet those costs, but that feathering rate *per se* may be a useful welfare indicator.

Second, the UV reflective properties of the skin and feathers of birds also play a role in social signalling (Passerines: Bize et al., 2006; Doucet and Montgomerie, 2003; Henderson et al., 2013; Keyser and Hill, 2003; Sirkiä and Laaksonen, 2009 Psittaciformes: Griggio et al., 2010) and have been found to correlate with reproductive success and corticosterone levels in another bird species (*Cyanistes caeruleus*: Henderson et al., 2013). Therefore, beyond the potential thermoregulatory or protective benefits of feathering to the individual, further research should explore social implications of enhanced feathering rate under UVA lighting.

Third, feather condition is also maintained through preening, and studies have demonstrated an increase in preening behaviours in chickens provided with UVA wavelengths (Kristensen et al., 2007; Ruis et al., 2010). The appearance of birds’ feathers in the presence of UVA wavelengths may provide more accurate cues of plumage condition than standard lighting, stimulating more preening behaviours. In budgerigars UV reflectance is lower in birds prevented from preening, and females spent more time with males with a higher UV reflectance in preference tests (Griggio et al., 2010; Zampiga et al., 2004). In light of our results, a further fruitful line of welfare research may be the relationship between UVA exposure, preening and feather condition.

Broiler chickens have been artificially selected for fast growth rates, though still retain UV reflective feathering which affects mate choice (Jones et al., 2001). It is possible that similar UV reflectance signals could exist in broiler chickens with applications for welfare assessment. The extent to which feathering acts as a social signal or indicator of condition in broiler chickens may in turn have other potential welfare implications. If, for example, an individual bird perceives that the majority of its flock-mates are in poor condition (potentially indicating a challenging environment), this could indicate to the bird they are living in an environment of poor quality. In contrast, if the majority of flock-mates appear to be in good condition (potentially indicating a good environment) this may be used as a cue that the environment is good quality.

The effect of UVA provision, or other husbandry changes that improve welfare, on aspects of plumage quality and feather directed behaviours in young broilers would be a promising area for further study.

### 4.2 Fearfulness

Broiler chickens provided with UVA exposure for the full 18-hour photoperiod were less fearful than control broilers, as indicated by shorter tonic immobility durations (Gallup, 1979). There was a non-significant trend for broilers provided with UVA + UVB for only 8 h a day to be less fearful than control broilers. This smaller effect may reflect a dose-dependent effect of UVA, resulting from an experimental limitation of UVB (hence also UVA) to 8 h a day, in treatment B. Contradictory impacts of UVB on T.I duration cannot be ruled out, though no studies currently support or refute this possibility. T.I duration was not affected by weight in the current study, indicating heavier birds (with reduced mobility) were not more fearful than lighter birds.

The impacts of UVA observed here are in agreement with findings by Ruis et al. (2010) and Maddocks et al. (2001) which support the idea that UVA reduces fearfulness. The provision of UVA may potentially eradicate fear and stress associated specifically with the ambiguity of visual feedback in environments lacking UVA, making it a valuable form of EE.

Other forms of EE that promote the expression of natural behaviours such as appropriate litter provision (Brantsæter et al., 2017; Pichova et al., 2016; Shao et al., 2015), elevated platforms (Norring et al., 2016) or providing straw bales (Kells et al., 2001) have been shown to reduce fearfulness and improve welfare. UVA wavelengths may potentially enhance the appearance of, or increase engagement with, other forms of EE leading to other indirect effects of UVA on fear reduction. UVA bulbs may represent an appealing enrichment option for commercial farms, who may be more likely to make one-off investments into UVA bulbs than other forms of EE associated with greater labour, time and floor space requirements.

### 4.3 Walking ability

Walking ability, assessed using the Bristol gait score criteria, was improved in both UV treatments. Heavier birds were more likely to obtain worse gait scores, which is consistent with the expectation that carrying more weight should impact on mobility, and results of previous studies (Kestin et al., 2001; Kristensen et al., 2006; Sørensen et al., 1999; Su et al., 1999). However, there was also an interaction between weight and treatment, with heavier birds in the UV treated groups having better gait scores than control broilers of similar weights.

Bailie et al. (2013) found the provision of natural light including UVA wavelengths, improved gait scores and increased latency to lie times in broiler chickens. However, their study design did not allow for distinction between which elements of natural light (wavelength composition or light intensity) were responsible for the results obtained. A study by Kristensen et al. (2006) found no improvements in gait score where UVA was provided, though the main light sources used in the study were fluorescent lights with spectral compositions distinct from the LEDs that were the main light source in the current study.

There is evidence to suggest UVA may increase activity and exploratory behaviours in chickens (Bailie et al., 2013; Kristensen et al., 2007; Maddocks et al., 2001; Ruis et al., 2010). Mechanical loading is essential for the normal development of bones and tendons, and decreased activity can negativly impact these tissues and consequently the walking ability of broiler chickens (Foutz et al., 2007a, 2007b; Moussa et al., 2007).

UVB light allows for the endogenous production of vitamin D and has been found to improve bone mineral density and reduce the incidence of tibial dyschondroplasia and rickets (Edwards, 2003; Fleming, 2008).

The improvements in gait score of UV treated broilers observed in the current study could potentially result from increased activity levels as a result of UVA wavelengths, or the provision of a localised area of 30 mw/cm² UVB may have been sufficient to support endogenous vitamin D production and skeletal growth.

The Bristol gait score is a subjective method of gait assessment where, based on a “snap-shot” of observed walking behaviour, a score is assigned to reflect good or impaired walking ability. The resulting output is influenced by many separate components of the broiler chickens integrated locomotor system; including the conformation and integrity of the skeleton, muscles and connective tissues essential for movement together with the central and peripheral nervous system which controls locomotion (motor neurons) and responds and adapts to mechanical and sensory feedback (sensory neurons).

Therefore, while improvements in Bristol gait score may reflect improved walking ability, it is still difficult to separate these integrated components of the locomotory system and determine precisely how husbandry manipulations such as wavelength composition affect walking ability.

### 4.4 Further discussion

The relationship between measures of fear, stress and feather condition is not well understood and it is interesting to note the relationship between feather score and tonic immobility duration is not consistent in both UV treated groups.

Previous studies in mature laying hens have found associations between poor plumage condition and higher levels of fear (Adams et al., 1978; Lampang and Craig, 1990; Mahboub et al., 2004) which is in agreement with the results obtained in the UVA only treatment of the current study. However, broiler chickens in the UVA + UVB treatment showed a non-significant trend to be less fearful and yet have poorer plumage than controls, which has also been reported by Campo et al. (2001).

Previous studies have assessed feather cover in laying hens at 40 (Adams et al.,1978), 72 (Campo et al., 2001), 57–60 (Lampang and Craig, 1990) and 20–48 (Mahboub et al., 2004), weeks of age. Thus, these scores likely reflect the direct impacts of environmental stressors or feather pecking rather than feather growth and development, which was assessed in the current study on broilers at 24 days old. UVA may have different impacts on the plumage condition and feather directed behaviours of mature broilers.

A possible explanation for the inconsistency in findings in the UV treated groups could be the UVA exposure time required to reduce fearfulness is less than the exposure time required to improve feather condition.

The links between feather growth, preening behaviours, UV reflectance, social interactions and levels of stress and fear with and without UV provision would be a promising area for further study in broiler chickens.

## 5 Conclusion

The provision of UV wavelengths, particularly UVA, has potential to improve the welfare of indoor reared broiler chickens. Lighting environments designed with the avian visual system in mind may improve the quality of visual feedback and reduce fearfulness. Future research identifying the links between UVA exposure and positive impacts on feathering rate, stress, activity levels and flock interactions would be of further importance to broiler welfare.

## Declarations of interest

None.

## With thanks to:

Neil Saunders, Jen Goodman, Kimberly Wilhite, Abril B. Izquierdo, Giuliana P. Miguel, Emily Fong, Ralph Hourd, Leigh Silvester, Ruth Wilcox, Hayley Smith, Vivian Arguelles, Lara Painter, Amey Brassington, Miroslaw Kasprzak, Chantelle Whelan and the BSU staff at the University of Nottingham who helped with the experiments. Further thanks are extended to Greengage Lighting Ltd. for supplying and installing the LED systems.
